# Improving care after hip fracture: the fracture? Think osteoporosis (FTOP) program

**DOI:** 10.1186/1471-2318-13-130

**Published:** 2013-12-05

**Authors:** Naomi Dore, Courtney Kennedy, Pauline Fisher, Lisa Dolovich, Leonardo Farrauto, Alexandra Papaioannou

**Affiliations:** 1 Hamilton Health Sciences-St. Peter’s Hospital, 88 Maplewood Avenue, Hamilton, ON L8M 1W9, Canada; 2 Department of Clinical Epidemiology & Biostatistics, Faculty of Health Sciences, McMaster University, Hamilton, Canada; 3 Department of Medicine, Faculty of Health Sciences, McMaster University, Hamilton, Canada; 4 Department of Family Medicine, Centre for Evaluation of Medicines, St. Joseph’s Healthcare, McMaster University, Hamilton, Canada; 5 Hamilton Health Sciences-Juravinski Hospital, Hamilton, Canada

**Keywords:** Hip fracture, Osteoporosis, Care gap

## Abstract

**Background:**

Hip fractures are a common and serious consequence of osteoporosis, and hip fracture patients are at high risk for recurrence. Appropriate pharmacotherapy reduces this risk and is associated with reduced mortality after hip fracture, but a care gap exists for fracture prevention in these patients. This evaluation determined rates of osteoporosis treatment and bone mineral density (BMD) testing in hip fracture patients following discharge from a rehabilitation unit.

**Methods:**

A prospective cohort study of hip fracture patients aged ≥ 50 on an inpatient rehabilitation unit in 2008 and 2011. Patients were seen by a nurse specialist, and encouraged to see their family physician for further assessment and treatment. Physicians were sent a letter indicating the need to follow up with their patient. Patients were contacted following discharge from hospital to determine treatment rates.

**Results:**

Of 310 eligible hip fracture patients admitted to the rehabilitation unit in the years studied, 207 patients were reached post-discharge and provided data. Of patients who were not previously taking osteoporosis medication, 59% of patients from the 2008 cohort, and 42% of patients from the 2011 cohort had osteoporosis treatment initiated by six months following discharge. By 2 months following discharge, 46% of patients in the 2008 cohort had a new BMD performed or scheduled, while this was true for 14% of patients from the 2011 cohort. 35% of patients in 2011 had not seen their family physician by 2 months following discharge.

**Conclusions:**

Rates for osteoporosis treatment and BMD testing were higher than those reported in the literature for patients not enrolled in case manager programs. BMD testing declined from 2008 to 2011. Lower treatment rates may be due to concerns regarding reports of possible association between bisphosphonate use and atypical fractures. Improving rates of patient follow-up with family physicians will be important for increasing hip fracture treatment rates after discharge.

## Background

Hip fractures are a common and serious consequence of osteoporosis. In 2007, the number of hospitalizations for hip fracture in Canada exceeded 28 000 [1]. Hip fractures are associated with significant morbidity and mortality, as well as a high cost to the health care system. The one-year mortality rate after hip fracture in Canada has been shown to be as high as 25% [2]. In addition, patients experience significant morbidity and loss of function after hip fracture, with an estimated 60% of patients requiring assistance and 33% totally dependent or in a long-term care facility a year later [3,4]. Canada spends an estimated $1.9 billion yearly on the treatment of osteoporotic fractures, which is expected to rise to $2.4 billion for hip fractures alone by the year 2041 [5]. Of all incident fractures, hip fractures have been found to have the highest excess, which are similar in relative magnitude to the cost of coronary heart disease and stroke [6].

Patients with hip fracture are at high risk for recurrent fracture; and have been reported to be at 1.9-2.5 times more likely to experience another fracture, depending on the location of the subsequent fracture [7]. Appropriate pharmacotherapy, however can substantially reduce the risk of fracture. The risk of vertebral fracture can be reduced by 30-70% depending on the therapy and level of adherence; the reduction in risk of non-vertebral fractures varies by fracture site [7]. In addition, bisphosphonates have all been associated with reduced mortality after hip fracture [8-11]. Current Canadian osteoporosis clinical practice guidelines identify patients with prior hip fracture as high risk for future fractures and recommend that these patients are investigated with a bone mineral density (BMD) and offered appropriate pharmacotherapy and lifestyle recommendations [8]. Alendronate, risedronate, zoledronic acid, and denosumab are first-line therapies for the prevention of hip, vertebral and non-vertebral fractures in post-menopausal women, and alendronate, risedronate and zoledronic acid and denosumab are first-line therapies for the prevention of fractures in men [8]. Despite the availability and benefit of these therapies, a care gap between recommendations and practice has been identified in Canada for fracture prevention in patients who have had a fracture, with fewer than 20% of women and 10% of men receiving pharmacotherapy to prevent further fractures [12-14].

In 2006 an initiative known as ‘Fracture? Think Osteoporosis’ (FTOP) was launched in Hamilton, Ontario with the aim of reducing subsequent fractures in patients presenting with fragility fractures, by improving recognition and treatment of osteoporosis in these patients. As part of this program patients presenting with fragility fracture were assessed in hospital and followed after discharge by a clinical nurse specialist acting as a case manager. Using an osteoporosis case manager has previously been shown to be a cost-effective way to substantially improve osteoporosis treatment rates and the quality of osteoporosis care after hip fracture during acute care hospitalization compared to usual care or educational initiatives alone [15-18]. Few programs, however, have focused on the rehabilitation population.

The purpose of this study was to examine the rate of appropriate post-fracture pharmacotherapy within six months of discharge in hip fracture patients admitted to rehabilitation during 2008 and 2011. A secondary objective was to examine the number of patients who had a BMD scheduled or performed and whether treatment initiation was related to ordering a BMD.

## Methods

### Design

This was a prospective cohort study.

### Patients

Patients aged 50 years or older who had a hip fracture and were inpatients at the rehabilitation unit of a tertiary-care hospital in Hamilton, Ontario in the years 2008 and 2011 were included in this study. This allowed comparison over time of care provided to patients involved in the FTOP program. Rehabilitation unit admission criteria included medically stable patients with clearly defined and attainable rehabilitation goals understood and agreed upon by the patient, with an estimated time to achieve goals ≤21 days, and able to physically tolerate participation in an active treatment program (1 hour + sitting tolerance twice daily; weight bearing; initial tolerance for physical activity for ½ to 1 hour three times daily).

### Description of the FTOP program

The FTOP program is a chronic disease management program for osteoporosis and consequent fractures in Hamilton. The objective of this multi-faceted interdisciplinary program is to improve diagnosis and treatment of osteoporosis in patients with fragility fractures and to decrease the rate of subsequent fractures in those individuals greater than 50 years who have experienced a fracture. Fragility fracture was defined as fracture that occurs spontaneously or following minor trauma such as a fall from standing height or less [8]. FTOP is a Hamilton Health Sciences initiative to reduce hip, spine and wrist fractures. The program included education and academic detailing by local osteoporosis leaders for rehabilitation and geriatric teams with respect to osteoporosis diagnosis and treatment in the immediate post-fracture period.

All hip fracture patients receive standardized orders which include calcium carbonate 500 mg and vitamin D 2000 IU daily in the inpatient hospital setting on acute care orthopedic units. Then, patients are transferred to the rehabilitation unit following their acute phase of care. Fragility hip fracture patients are assessed by the clinical nurse specialist on the rehabilitation ward. The clinical nurse specialist provides education to the patient regarding osteoporosis, falls, and appropriate nutritional and supplement intake. The patient is also given a “fracture alert” form (see Additional file 1), that indicates that they should follow up with their family physician for further assessment, including bone mineral density (BMD) testing and consideration of treatment for osteoporosis. A letter (see Additional file 2) is also sent to the patient’s family physician suggesting that they follow up with their patient following discharge from hospital for further assessment and treatment for osteoporosis.

Two months after discharge from hospital, hip fracture patients receive a phone call from the clinical nurse specialist to determine if they have had a BMD and if they are now on treatment for osteoporosis. At that time, further education is provided with regard to osteoporosis and treatment, and if necessary, the need to follow up with their family physician. If patients are not on treatment for osteoporosis at the 2-month follow up, they are given a further follow up call six months after discharge. Only one clinical nurse specialist was involved in the study and provided the patient assessments, education and follow-up, and sent out fracture alert letters to physicians for both the 2008 and 2011 patient cohorts.

Patients with fractures secondary to malignancy were excluded. Patients were also excluded if they were transferred to an outside facility for care.

### Data collection

Data obtained included: age, gender, fracture type/location, diagnosis of osteoporosis on chart, knowledge of osteoporosis diagnosis, if taking calcium supplement and dose, amount of dietary calcium, if taking vitamin D supplement and dose, if taking osteoporosis medication and if using appropriately as assessed by nurse, osteoporosis medications started in hospital, seen family doctor since discharge, BMD prior to admission, BMD completed or scheduled since discharge, falls and fracture (and fracture type/location) since discharge. A database containing de-identified data was used for analysis. Care provided including treatment in-hospital was confirmed by the patient chart and care initiated post-discharge was collected by patient report.

### Outcome variables

The primary outcome was initiation of osteoporosis treatment (i.e. post-discharge) by 6-months. Medications considered were: any oral bisphosphonate, zoledronic acid, teriparatide, denosumab.

The secondary outcome was a new BMD performed or scheduled since discharge (patient self-report). Since only patients who were not on treatment at 2-months were contacted at 6-months, numbers are reported for both the 2- and 6-month time periods.

### Statistical analysis

Continuous variables were summarized using mean, standard deviation and categorical variables were summarized as percentages. Between-group comparisons were performed using Pearson chi-square and independent samples t-tests.

Statistical significance was defined as p < 0.05. Statistical analyses were performed with SPSS software 20.0® (SPSS Inc., Chicago IL).

### Ethical considerations

No formal consent process was used as data collected was considered part of regular care. The study was approved by the Hamilton Health Sciences Research Ethics Board.

## Results

**Figure 1:**
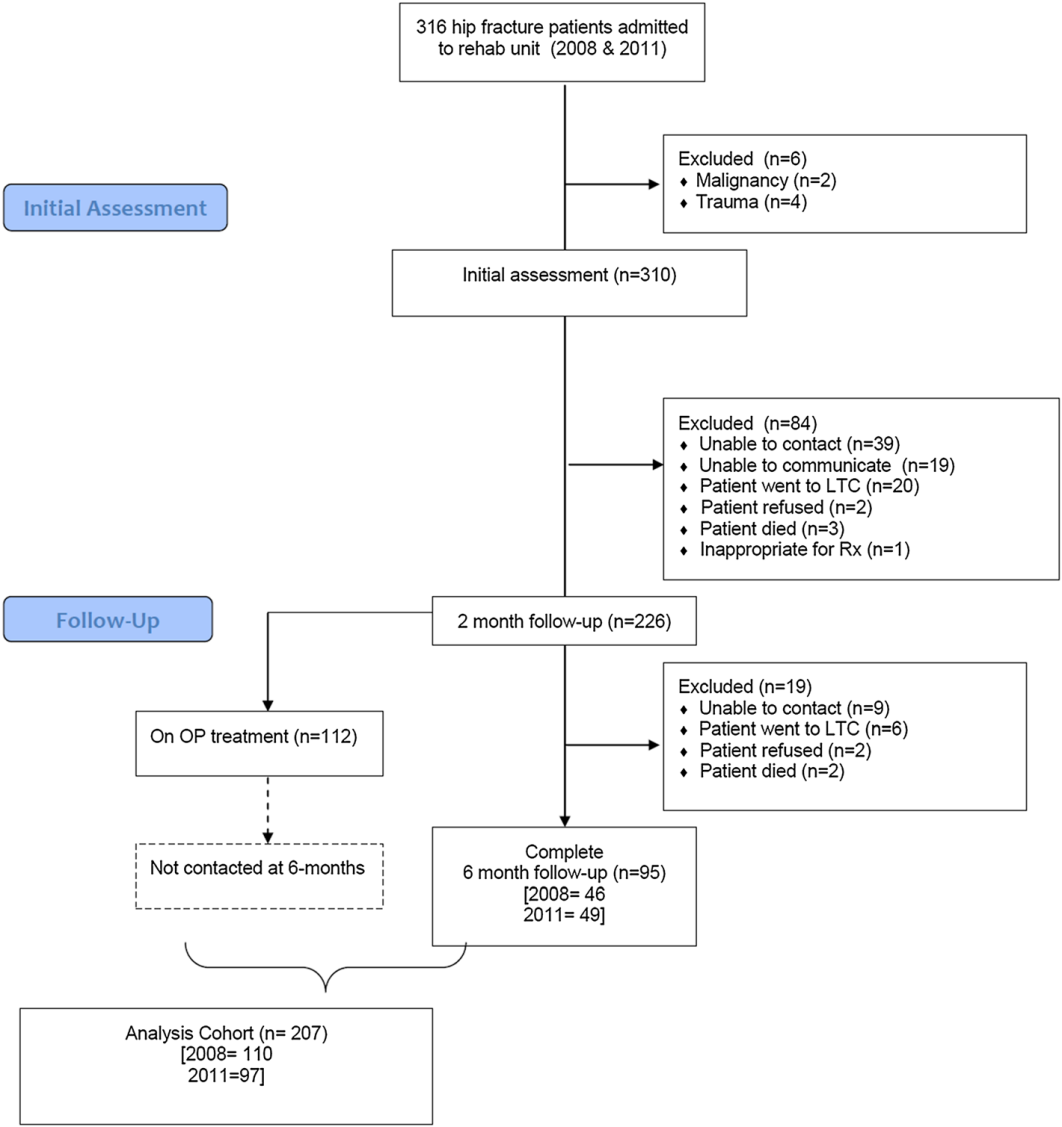
**Flow diagram.** OP = osteoporosis and LTC = long-term care.

A total of 821 hip fracture patients presented at the teaching hospital in the two years studied (439 in the year 2008 and 382 in the year 2011). Of these patients, 316 (38%) were transferred to the rehabilitation unit. The remaining patients were transferred to their community hospital (n = 94), other rehabilitation hospital (n = 6), chronic care hospital (n = 18), home care program (n = 83), home for the aged or nursing home (n = 181), home (n = 52), ambulatory out-patient clinic (n = 1), other unclassified health institution (n = 4), or signed out (n = 2) or died (n = 64). As described in Figure 1, of 316 hip fracture patients transferred to rehabilitation, 310 had fragility fractures and were eligible for study (153 from the 2008 cohort, and 157 from the 2011 cohort). After excluding patients unable to be reached for the 2-month or 6-month follow-up calls, a total of 207 patients made up the final analysis cohort.

The mean age of patients analyzed from 2008 and 2011 was 81.5 years, ranging from 52–104 years. There were 150 females and 57 males. At the time of initial assessment, 56% of patients from 2008 and 52% of patients from 2011 had a diagnosis of osteoporosis documented in their chart (Table 1).

**Table 1:** Baseline demographics and clinical characteristics, % (n)

	**2008**		**2011**	
	**All patients** **(n = 153)**	**Analysis cohort** **(n = 110)**	**All patients** **(n = 157)**	**Analysis cohort** **(n = 97)**
Age in years, mean (SD)	80.8 (8.82)	80.7 (8.93)	82.1 (9.36)	82.3 (9.34)
Female	73% (112)	72% (79)	69% (109)	73% (71)
Previous diagnosis (patient self-report)	37% (56)	40% (44)	24% (38)	34% (33)
Diagnosis OP in chart	52% (79)	56% (61)	46% (72)	52% (50)
OP treatment prior to fracture	33% (50)	40% (44)	22% (35)	29% (28)
Recent BMD* prior to fracture	31% (48)	35% (38)	17% (27)	22% (21)
Vitamin D supplement use prior to fracture	53% (81)	60% (66)	58% (91)	67% (65)
Calcium supplement use prior to fracture	54% (82)	60% (66)	53% (83)	62% (60)
Patient has heard of OP	80% (122)	83% (91)	66% (103)	77% (75)

*For the 2008 cohort, this was in the past 1-year; for the 2011 cohort, the past 2-years.

OP = osteoporosis.

### Calcium and vitamin D

By six months post-discharge from rehabilitation, 93% (102/110) of patients from the 2008 cohort and 81% (79/97) of patients from the 2011 cohort were taking calcium supplementation (≥500 mg). 94% (103/100) and 93% (90/97) of patients were taking vitamin D supplementation (≥800 IU) in 2008 and 2011, respectively. Please refer to Table 2.

**Table 2:** Percent (n) of patients with osteoporosis pharmacotherapy within 6-months of discharge

	**2008** **(n=110)**	**2011** **(n=97)**
Taking OP medication by 6-months	75%	56%
	(82/110)	(54/97)
Initiated after involvement in FTOP^a^	35%	30%
	(39/110)	(29/97)
Taking at baseline	n = 44	N = 28
Discontinued treatment after baseline	n = 1	n = 3
Taking vitamin D supplement (≥800 IU) by 6-months^b^	94%	93%
	(103/110)	(90/97)
Initiated after involvement in FTOP^c^	36%	28%
	(40/110)	(27/97)
Taking at baseline	n = 66	N = 65
Discontinued treatment after baseline	n = 3	n = 2
Taking calcium supplement (≥500 mg) by 6-months^b^	93%	81%
	(102/110)	(79/97)
Initiated after involvement in FTOP^d^	36%	26%
	(40/110)	(25/97)
Taking at baseline	n = 66	60
Discontinued treatment after baseline	n = 4	n = 6

^a^Patients were not previously taking osteoporosis medication. (OP = osteoporosis).

^b^For patients who were not contacted at 6-months (because they taking osteoporosis treatment at the 2-month assessment), the answer the provided regarding supplementation at 2-month assessment was used.

^c^Patients not taking any vitamin D prior to FTOP.

^d^Patients not taking any calcium prior to FTOP.

### Initiation of pharmacotherapy

Overall 75% (82/110) of patients from the 2008 cohort and 56% (54/97) of patients from the 2011 cohort were taking osteoporosis medication 6 months after discharge from rehabilitation (Table 2). Of patients who were not previously taking osteoporosis medication, 59% (39/66) of patients from the 2008 cohort and 42% (29/69) of patients from the 2011 cohort had treatment initiated by 6 months after discharge from rehabilitation.

### Bone mineral density testing

By 2 months post discharge from rehabilitation, 46% (54/118) of patients in the 2008 cohort had a new BMD performed or scheduled. Only 14% (15/108) of patients in the 2011 cohort had a new BMD performed or scheduled by the 2-month follow-up. Of the patients who were not on treatment at 2-months and could be contacted again at 6-months (Figure 1; n = 46 for 2008 and n = 49 for 2011), 61% (2008) and 20% (2011) had a new BMD performed or scheduled.

In the 2008 cohort at 2 months post discharge from rehabilitation, no difference was found in the BMD status of those who were taking osteoporosis medication compared to those who were not. However, in the 2011 cohort, 23% of patients taking osteoporosis medication by 2 months post discharge had a new BMD ordered since involvement in FTOP, compared to 7% of those not on treatment (p = 0.015). In addition 44% of patients being treated with osteoporosis medication by the 2-month post discharge mark had a recent BMD done in the last 1–2 years, compared to only 23% of non-treated patients (p = 0.024).

### Falls and fractures during follow-up

From the 2008 cohort, 13% (15/118) of patients had fallen at least once by 2 months post discharge from rehabilitation, and there were 5 subsequent fractures (4 hip fractures, and 1 shoulder fracture). From the 2011 cohort, 15% (16/108) of patients reported to have fallen at least once by 2 months post discharge, with 2 subsequent hip fractures.

## Discussion

This study of hip fracture patients in the rehabilitation setting demonstrated that involvement in the FTOP case manager and academic detailing program resulted in higher osteoporosis treatment and BMD rates than those reported in the literature for patients not involved in similar types of case-manager led programs [12-14,19]. A systematic review of 37 studies on post-fracture osteoporosis diagnosis and treatment found osteoporosis treatment rates ranging from 0.5% to 38%, but only six studies reported treatment rates greater than 10% [19]. In our study of patients involved in FTOP, among patients with no previous osteoporosis treatment 50% had osteoporosis treatment initiated by 6 months post discharge. These rates are consistent with previous studies of case manager osteoporosis programs [15,20,21]. For example, a randomized controlled trial evaluating a case manager osteoporosis program in acute care for hip fracture patients demonstrated improved rates of osteoporosis treatment in patients involved in the program, with treatment initiated in 51% of these patients [15]. Our study demonstrated that 60% of patients had a diagnosis of osteoporosis documented in their chart, which is an improvement on previously reported rates of post-fracture osteoporosis diagnosis of less than 30% [12]. A history of prior fracture rate in our patients (46%) was consistent with literature from other settings, highlighting prior fracture as an important risk factor for future fracture [12,13,15,20].

Interestingly, we found no improvement in osteoporosis treatment rates and BMD referral in 2011 compared to three years earlier. In fact, we observed a decline in post-fracture BMD testing from 2008 to 2011, which is a concern considering BMD testing has been associated with treatment initiation [22,23]. A possible explanation for this decline in BMD testing could be the implementation of the 2010 clinical practice guidelines for osteoporosis care in Canada. These guidelines recommend that individuals over the age of 50 who have had a fragility fracture of the hip are automatically at high risk of future fracture and should be offered pharmacologic therapy, regardless of BMD [8]- although we did not see the increase in treatment rates one would expect if this guideline was being followed. In fact, data from the 2011 cohort did show that those patients who received a recent BMD or had a new BMD ordered since involvement in FTOP were more likely to receive osteoporosis treatment.

Unlike some previously reported case manager programs, our program did not refer patients to a specialty clinic, but rather relied on primary care physicians for patient care. A study that surveyed 1000 Ontario family physicians found that over 80% of physicians wanted to be more informed about osteoporosis management, and that the importance of a prior fracture in the management of osteoporosis was not well known by most family physicians [24]. The FTOP program aimed to improve this by sending out alert letters to the patients’ family physicians, incorporating a reminder and educational information about post- hip fracture osteoporosis management. Systematic review has previously found this strategy to be effective in increasing BMD testing and osteoporosis medication use [25]. However, patients can only benefit if they are actually seen by their family physician; and our study found that in the 2011 group, 33% of patients had not seen their family physician by 6 months after discharge from rehabilitation.

Standardized orders for calcium and vitamin D in acute care for patients with fractures were in place at the hospital for both 2008 and 2011 cohorts. The high rates of calcium and vitamin D use in our patients in hospital (92% and 96% respectively in the 2011 cohort) would suggest these have been integrated into routine clinical practice. In contrast, it is clear that the number of patients being treated with other osteoporosis medication in hospital has declined, as 81% of patients did not have osteoporosis treatment ordered in hospital in 2011. In comparison, a previous study found that 56% of patients transferred to a rehabilitation or a geriatrics ward between 2003 and 2004 were started on a bisphosphonate during index admission [20]. A possible contributor to this decline in treatment initiation could be the emergence of reports about atypical fractures associated with bisphosphonate use, although this has since been shown to have low incidence. One study demonstrated the risk of proximal femur fractures with bisphosphonate therapy to be 2.3 per 10 000 patient years [26].

Our results are consistent with the findings of a systematic review of studies evaluating osteoporosis diagnosis and/or treatment post-fracture, where the proportion of patients diagnosed or treated for osteoporosis appeared to be greater in follow-up studies than at discharge [12]. However, success has been reported with a post-fracture osteoporosis education and treatment program pre-discharge, with >95% of patients appropriately diagnosed and treated, but to accomplish this required the full cooperation of orthopaedic surgeons and residents, orthopaedic technologists, allied health-care professionals and administrative staff in addition to a dedicated coordinator [17].

There are limitations to this study that should be acknowledged. The sample was limited by the number of patients who were admitted into rehabilitation (n = 316), and by the substantial number of patients with whom we were unable to follow-up after discharge from rehabilitation (n = 103) for various reasons (Figure 1). Given the small sample size, our analysis was limited to mainly descriptive statistics. The results from this rehabilitation population may not be applicable to other patient groups, for example those in long term care or home care programs. The results post-discharge from rehabilitation are based on patient self-report. In this elderly population, patients may be unfamiliar with the medication they take and recall bias may be an issue for reported use of osteoporosis treatment as well as calcium and vitamin D. Adherence is a known issue with osteoporosis medication; research suggests that approximately 20-30% of osteoporosis patients will stop taking their bisphosphonates, raloxifene or HRT by 12 months after these medications were prescribed [27]. Adherence was not measured in this study, and patients treated at 2 months post-discharge were assumed to be still on treatment at the six month mark. We did not contact patients at 6-months if they were taking treatment at the 2-month follow-up, thus our analysis cohort was limited for examining BMD variables at 6-months.

## Conclusions

Future research should focus on better integration between primary care physicians and hospitals to improve osteoporosis treatment rates for hip fracture patients post-discharge from rehabilitation. Ensuring that patients follow-up with their family physicians has the potential to impact mortality in hip fracture patients. The mean age of patients in our sample was 82; many physicians have uncertainties around applying treatment guidelines to older patients with comorbidities and polypharmacy. There is a need to educate both patients and health care professionals around treating older patients after hip fracture. Our team is currently updating the 2010 Canadian Osteoporosis Guidelines with a focus to closing the care gap and reducing the risk of future fracture in high risk populations such as elderly populations with hip fracture.

## Competing interests

Alexandra Papaioannou is or has been a consultant, or on a speaker’s bureau, or received unrestricted grants from Amgen, Eli Lilly, Merck Frosst Canada, Novartis, Warner Chilcott; she has also conducted clinical trials for Eli Lilly, Merck Frosst, Novartis and Pfizer. Naomi Dore, Courtney Kennedy, Pauline Fisher, Lisa Dolovich, and Leonardo Farrauto declare that they have no competing interests.

## Authors’ contributions

ND participated in the data analysis, literature review and manuscript writing. CK assisted with the data analysis and manuscript editing. PF participated in the data collection, assisted with data analysis and manuscript editing. LD participated in the study concept, assisted with data analysis and manuscript editing. LF participated in the project development and implementation. AP participated in the project development and implementation, study concept, data analysis and manuscript editing. All authors read and approved the final manuscript.

## Pre-publication history

The pre-publication history for this paper can be accessed here:

http://www.biomedcentral.com/1471-2318/13/130/prepub

## Supplementary Material

Additional file 1 Fracture alert form. Click here for file

Additional file 2 Fracture alert to general practitioner. Click here for file
