# Supplementary material for: Improving care after hip fracture: the fracture? Think osteoporosis (FTOP) program
Source: BMC Geriatr. 2013 Dec 5;13:130. doi: 10.1186/1471-2318-13-130 (PMC4029576; doi:10.1186/1471-2318-13-130)
Supplement: Additional file 1 — Fracture alert form. [file 1471-2318-13-130-S1.docx]

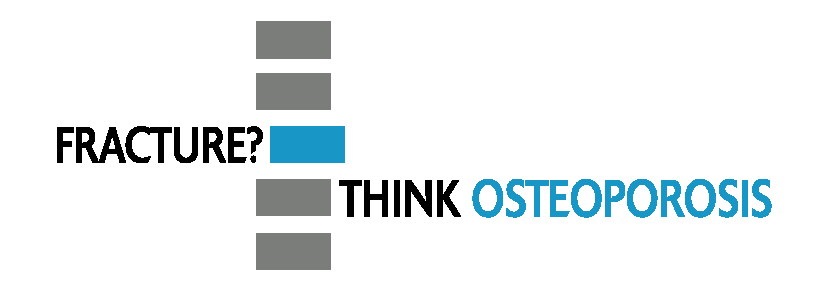


##### Osteoporosis Fracture Alert

If you are over 50 and you have broken your:

- wrist
- hip
- a bone in your back or spine

**you may have Osteoporosis.**

Osteoporosis is when your bones become thinner and weaker. This means that you have a greater chance of breaking another bone.

**Please make an appointment with your family doctor to talk about:**

- getting tested and treated for osteoporosis
- how much Calcium and Vitamin D is in the food you eat, and
- what medications and supplementation supplements you need to take to prevent a fracture.

To help prevent another broken bone, everyday you need to:

- get enough Calcium – 1200 mg (diet and supplements
- get enough Vitamin D –1000 – 2000 IU daily
- exercise
- prevent a fall

#### **People with healthier bones live better lives**

For more information contact

Osteoporosis Canada

1-800-463-6842

www.osteoporosis.ca

| ©Hamilton Health Sciences  October, 2012. | 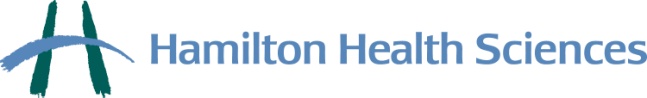 |
| --- | --- |
