# Supplementary material for: Improving care after hip fracture: the fracture? Think osteoporosis (FTOP) program
Source: BMC Geriatr. 2013 Dec 5;13:130. doi: 10.1186/1471-2318-13-130 (PMC4029576; doi:10.1186/1471-2318-13-130)
Supplement: Additional file 2 — Fracture alert to general practitioner. [file 1471-2318-13-130-S2.docx]

|  |  |
| --- | --- |

##### Fracture Alert

Dear Dr. _______________________	Date: ______________________

Your patient ___________________________ has presented to Hamilton Health Sciences with a hip fracture.

Further assessment or treatment for Osteoporosis is recommended:

- Bone Mineral Density (BMD) test. (BMD testing should take place on the same machine as any previous BMD Tests completed)

Please consider at a minimum:

| - Calcium 1200 mg per day via diet or supplements |  |
| --- | --- |
| - Vitamin D 1000 – 2000 IU per day |  |

Treatment to prevent the next fracture:

| Bisphosphonates: | Alendronate (Fosavance®) 70 mg weekly  Alendronate (Fosamax®) 70 mg weekly  Risedronate (Actonel®) 35 mg weekly or 150mg monthly  Zolendronic Acid (Aclasta® 5m mg IV annually |
| --- | --- |
| SERM: | Raloxifene (Evista®) 60 mg daily |
| Rank ligand inhibitor | Denosumab (Prolia®) 60 mg sc q 6 months |
| PTH: | Forteo ® 20 mcg sc. daily |
| Calcitonin: | Miacalcin 200iu nasal spray to alternate nares |

Please see attached new Osteoporosis guidelines.

**For consultation at the Osteoporosis and Fracture Prevention Clinic, please fax a referral to: 905-549-7003 or call 905‑521-2100 ext. 77248**

Sincerely,

Dr. Alexandra Papaioannou

M.D., M.Sc., F.R.C.P.(C), F.A.C.P.
